# Hospital mortality predictive factors following rapid response team activation in very old patients

**DOI:** 10.1186/cc14681

**Published:** 2015-09-28

**Authors:** Henrique Palomba, Antonio Capone, Felipe M de T Piza, Michelle Jaures, Thiago D Corrêa

**Affiliations:** 1 Hospital Israelita Albert Einstein, Morumbi, São Paulo, SP, Brazil

## Introduction

The number of very old (≥80 years) patients with chronic illness and functional impairment requiring major medical attention is increasing, with great chances of adverse events during hospitalization. Rapid Response Teams (RRT) were implemented to improve the recognition and response to deteriorating ward patients; however, the characteristics and outcomes of very old patients assisted by RRT during hospitalization are not well described.

## Objective

The primary objective was to evaluate the rate, characteristics and outcomes of very old patients seen by RRT during hospitalization.

## Methods

A total of 538 very old patients assessed by RRT between January 2012 and December 2013 were included. Multivariate analysis was used to evaluate which variables were associated with hospital mortality. Early RRT call was defined as RRT activation <48 hours from hospital admission and late RRT call if it happened >48 hours from hospital admission.

## Results

The mean age was 87 ± 4.8 years and 42% (*n *= 224) were male. There were 193 (36%) early calls and 345 (64%) late calls. The main reasons for RRT activation were respiratory failure in 35% (*n *= 186) and mental status changes in 15% (*n *= 80). The distribution of RRT activation was uniform over the 24-hour period, with 55% (*n *= 298) of calls during the day (7:00 a.m.-7:00 p.m.) and 45% (*n *= 240) overnight (7:00 p.m.-7:00 a.m.). A total of 153 patients (28%) were admitted to the ICU and the overall mortality rate was 20% (*n *= 110). The multivariate analysis showed the following variables as significantly associated with hospital mortality: late (>48 hours) RRT call (OR 1.73; 95% CI 1.07-2.79), acute change in oximetry saturation to <90% (OR 1.56; 95% CI 1.01-2.40) and admission to step-down unit (OR 0.48; 95% CI 0.25-0.91).

## Conclusion

In this study, hospital mortality predictive factors of very old patients seen by RRT during hospitalization were late RRT call and acute respiratory failure.

